# Influence of degree correlations on network structure and stability in protein-protein interaction networks

**DOI:** 10.1186/1471-2105-8-297

**Published:** 2007-08-09

**Authors:** Caroline C Friedel, Ralf Zimmer

**Affiliations:** 1 LFE Bioinformatik, Institut für Informatik, Ludwig-Maximilians-Universität München, Amalienstraße 17, 80333 München, Germany

## Abstract

**Background:**

The existence of negative correlations between degrees of interacting proteins is being discussed since such negative degree correlations were found for the large-scale *yeast *protein-protein interaction (PPI) network of Ito et al. More recent studies observed no such negative correlations for high-confidence interaction sets. In this article, we analyzed a range of experimentally derived interaction networks to understand the role and prevalence of degree correlations in PPI networks. We investigated how degree correlations influence the structure of networks and their tolerance against perturbations such as the targeted deletion of hubs.

**Results:**

For each PPI network, we simulated uncorrelated, positively and negatively correlated reference networks. Here, a simple model was developed which can create different types of degree correlations in a network without changing the degree distribution. Differences in static properties associated with degree correlations were compared by analyzing the network characteristics of the original PPI and reference networks. Dynamics were compared by simulating the effect of a selective deletion of hubs in all networks.

**Conclusion:**

Considerable differences between the network types were found for the number of components in the original networks. Negatively correlated networks are fragmented into significantly less components than observed for positively correlated networks. On the other hand, the selective deletion of hubs showed an increased structural tolerance to these deletions for the positively correlated networks. This results in a lower rate of interaction loss in these networks compared to the negatively correlated networks and a decreased disintegration rate. Interestingly, real PPI networks are most similar to the randomly correlated references with respect to all properties analyzed. Thus, although structural properties of networks can be modified considerably by degree correlations, biological PPI networks do not actually seem to make use of this possibility.

## Background

All biological processes of a cell such as proliferation, signal transduction or apoptosis are shaped by proteins interacting specifically with each other and building more or less transient complexes. To understand these processes, determining the underlying protein interactions is of vital importance. The advent of high-throughput methods such as yeast two-hybrid (Y2H) has made it possible to determine protein interactions on a large scale. This development has lead to the determination of several large-scale species-specific protein-protein interaction networks in the last years [1-7].

Apart from biological implications, protein-protein interaction networks are also analyzed for more general features such as topology, tolerance to attack or failure, local clustering and sampling effects due to measurement errors [8-16]. Protein-protein interaction networks belong to the class of so-called scale-free networks [8]. This means that the number of interactions of a protein, i.e. its degree, follows approximately a power-law distribution with many proteins forming only very few interactions and a few promiscuous proteins (hubs) forming many. As a consequence, protein interaction networks are vulnerable to a targeted attack selectively on hubs [17], but tolerant to random failure of nodes. Indeed, a correlation between the lethality of a protein knockout and the corresponding protein degree has been shown [8].

The degree distribution is the most commonly analyzed network characteristic. However, since the same number of connections can be formed in several ways with the same degree distribution, it does not characterize a network completely. For instance, low-degree nodes might associate preferentially either with other low-degree nodes or with hubs. Thus, two networks can have the same degree distribution and still differ in other aspects of network structure and react differently to perturbations. Correlations between degree values of neighboring nodes were analyzed by Maslov and Sneppen [18,19] for the *yeast *interaction network determined by Ito et al. [1]. Maslov and Sneppen found that interactions between hubs are significantly suppressed relative to a "null model" and that hubs tend to associate with low-degree nodes instead. Contrary to that, more recent studies [13,20] showed no such negative correlation between node degrees in *yeast *for high-confidence interaction sets. These contrasting results may be explained by a bias in the yeast-two hybrid system [21] which might artificially increase negative degree correlations. On the other hand, we cannot exclude the possibility that pooling data from various sources and restricting to high-confidence interactions might affect degree correlations as well.

In this article, we investigated to what extent degree correlations are present in experimentally derived PPI networks. For this purpose, we compared several PPI networks against reference networks showing no degree correlations (the "null model" of Maslov and Sneppen). Additionally, a model was developed to create reference networks with positive and negative correlations between the degrees of neighboring nodes, respectively. Based on simulations, we then evaluated how positive or negative degree correlations affect network structure and tolerance of the networks to targeted deletions.

We found that negative degree correlations lead to less fragmentation of the original networks into connected components compared to positively correlated networks. On the other hand, such negative correlations increase the vulnerability of the corresponding networks to targeted deletions of hubs. This can be seen from a higher rate of interaction loss and an increased disintegration rate in negatively correlated networks. Our results show that PPI networks tend to be most similar to the "null model" networks. Thus, significant modifications in network structure are possible without changing the degree distribution. However, only a very small range of modifications is realized in protein-protein interaction networks. Our results suggest that the mostly uncorrelated network structure of PPI networks might be a consequence of different selective disadvantages of both negatively and positively correlated networks.

## Results

### Protein-Protein interaction networks

For this article, the following protein-protein interaction networks were analyzed: (i) networks from yeast two-hybrid (Y2H) experiments, (ii) networks extracted from the Database of Interacting Proteins (DIP) [22] and (iii) the *yeast *high-confidence interaction set compiled by Batada et al. [20]. In the first case, we used results from the large-scale Y2H studies of Ito et al. [1] and Uetz et al. [2] for *yeast*, Giot et al. [4] for *drosophila*, Li et al. [3] for *C. elegans*, Rual et al. [5] and Stelzl et al. [6] for *human *and LaCount et al. [7] for *P. falciparum*. From DIP we used the species-specific data sets for *yeast*, *drosophila*, *human *and *E. coli *(see Table 1 for network characteristics).

**Table 1:** Network characteristics for PPI networks.

	|*V*|		*L*	# connected components	*r*	FER
**Rual et al.**	1549	3.37	4.36	118	-0.198	0.18
**Stelzl et al.**	1705	3.70	4.85	44	-0.191	0.16
**Ito et al. (complete)**	3279	2.68	4.88	195	-0.176	0.22
**Li et al. (core)**	1415	2.94	4.91	70	-0.176	0.19
***Yeast *(DIP)**	4959	6.95	4.15	31	-0.133	0.26
**Ito et al. (core)**	797	1.89	6.14	143	-0.112	0.35
**Uetz et al.**	1005	1.80	7.49	177	-0.088	0.40
***E. coli *(DIP)**	1840	6.44	3.80	346	-0.086	0.12
***D. mela*. (DIP)**	7451	6.08	4.39	62	-0.081	0.24
**Batada et al.**	2998	6.18	4.90	101	-0.047	0.37
**LaCount et al.**	1308	4.20	4.26	23	-0.025	0.31
***Human *(DIP)**	1085	2.48	6.77	126	-0.004	0.36
**Giot et al.(core)**	4651	2.01	9.43	591	0.023	0.46

This table shows several network characteristics for the protein-protein interaction networks. Shown are the number of nodes |*V*|, the average degree k¯
 MathType@MTEF@5@5@+=feaafiart1ev1aaatCvAUfKttLearuWrP9MDH5MBPbIqV92AaeXatLxBI9gBaebbnrfifHhDYfgasaacH8akY=wiFfYdH8Gipec8Eeeu0xXdbba9frFj0=OqFfea0dXdd9vqai=hGuQ8kuc9pgc9s8qqaq=dirpe0xb9q8qiLsFr0=vr0=vr0dc8meaabaqaciaacaGaaeqabaqabeGadaaakeaacuWGRbWAgaqeaaaa@2E23@, the characteristic path length *L*, the number of connected components, the correlation coefficient *r *and the fraction of edges remaining (FER) after deleting the 10% nodes with the highest degree. Networks are sorted according to correlation coefficient.

For the Li and Giot networks, only high-confidence interactions were considered. For the Ito networks, both the high confidence interaction set and the complete interaction set were analyzed separately to compare our results against the ones of Maslov and Sneppen. Interactions from different experiments for the same organism were analyzed individually in order to compare them against each other.

The original networks were compared against three types of reference networks which were created by rearranging the connections in the network such that each node has the same degree as before (see methods): negatively correlated references, positively correlated references and uncorrelated, rewired references (the "null model" of Maslov and Sneppen). The latter ones exhibit only random degree correlations given the degree distribution. For each PPI network, 100 uncorrelated, positively and negatively correlated reference networks were generated, respectively, and results were averaged over the 100 individual simulation runs.

### Degree correlations in PPI networks

**Figure 1:**
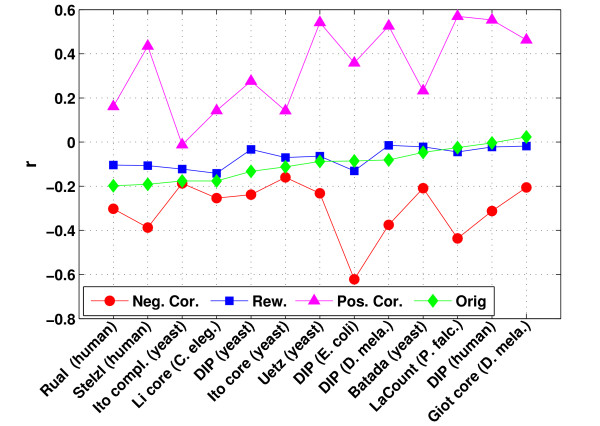
**Correlation coefficients**. This figure shows the correlation coefficients observed in the original PPI networks as well as in the corresponding negatively correlated, rewired and positively correlated reference networks (see methods). Networks are sorted according to the correlation coefficient in the original PPI networks.

The original PPI networks were compared against the three reference networks for the correlation coefficient *r *calculated between the degrees of connected nodes (see methods). This comparison showed that the original networks tend to have correlation coefficients similar to or slightly smaller than the uncorrelated "null model" networks (see Figure 1). Higher correlation coefficients than in the rewired networks are only observed for the *E. coli *and *human *interaction maps from DIP and the *P. falciparum *and *drosophila *Y2H networks. Interestingly, the PPI networks with the highest similarity to the negatively correlated reference networks are the complete *yeast *interaction set from Ito et al. and to a lesser degree also the Ito core set. This is consistent with previously reported results [18,19]. Contrary to that, the second large-scale *yeast *interaction set from Uetz et al. and the high-confidence network compiled by Batada et al. do not show a suppression of connections between hubs.

In general, the correlation coefficients of the rewired networks are also negative. This is a consequence of the positive skew in the degree distributions of the PPI networks (see methods for the definition of skewness and Additional file 1: Supplementary Figure 1) which leads to only few high degree nodes. As a consequence, hubs tend to be connected to low-degree nodes since those are most abundant even if connections between hubs are not suppressed. Differences in the degree distribution between the PPI networks also explain why the correlation coefficients in the positively and negatively correlated networks are in some cases close to the correlation coefficients of the rewired networks and in some cases far apart. Since large-scale experiments are very error-prone, we simulated the effects of measurement errors on the networks by randomly removing 10% of the interactions and adding another 10% in four different ways (see methods). The four different strategies for simulating measurement errors resulted in slight variations in the correlation coefficients of the PPI networks (see Additional file 1: Supplementary Figure 2). Nevertheless, the resulting correlation coefficients are still more similar to the rewired networks than to any of the reference networks.

### Structural properties influenced by degree correlations

**Figure 2:**
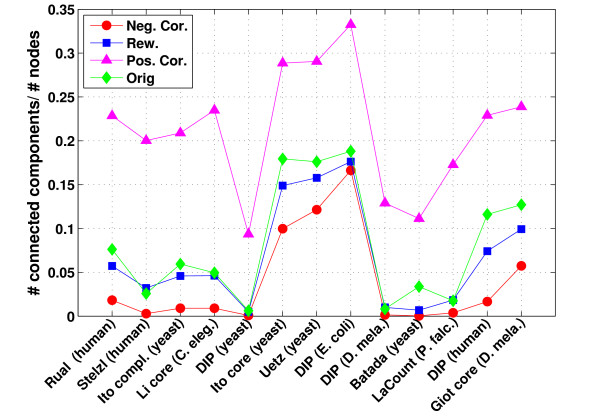
**Number of connected components**. For the PPI networks and the negatively correlated, rewired and positively correlated reference networks (see methods), the number of connected components is illustrated. Since networks sizes and consequently also the number of connected components vary greatly between networks, numbers were scaled by dividing by the original network size. The highest number of connected components are always observed in the positively correlated networks and the lowest number in the negatively correlated ones.

We calculated for the original and reference networks the number of connected components in the networks as well as the characteristic path length over all nodes and over hubs only (see methods). For the number of connected components significant differences can be observed between the different types of degree correlations in networks (see Figure 2). The number of connected components is highest in the positively correlated networks and lowest in the negatively correlated ones. Thus, positive correlations lead to an increased fragmentation of the network into separated clusters. Despite this trend, the PPI networks tend to consist of slightly more connected components than the rewired networks, even if they are characterized by smaller correlation coefficients than the latter.

For characteristic path length no consistent tendency can be observed (see Additional File 1:Supplementary Figure 3). In some cases positively correlated references have higher characteristic path lengths than negatively correlated references (e.g. the *human *network by Rual et al.). In some cases it is the other way around (e.g. the *yeast *network by Uetz et al.). If we restrict the calculation of the characteristic path length to paths between hubs (top 10% highest degree nodes), we observe the behavior expected from the degree correlations. The average distance between hubs is generally lowest for the positively correlated networks and highest for the negatively correlated networks (see Additional File 1: Supplementary Figure 3). Again, the protein-protein interaction networks tend to show characteristic path lengths between hubs similar to or slightly larger than the rewired networks. Despite the significant negative degree correlations observed in the complete and high-confidence interaction networks from Ito et al., these networks actually have significantly shorter path lengths between hubs than the negatively correlated networks.

Interestingly, in all original PPI networks and all reference networks, hubs tend to lie in the same largest component (also called the giant component). Accordingly, the number of hubs connected by a path is in general significantly higher than would be expected for a random selection of 10% of nodes from the network. On average, connections between hubs make up between 0.99% and 6.7% of connections between all nodes. These values are highest for the positively correlated networks which consist of more components than the other network types.

### Tolerance to targeted deletion

Apart from network properties, we compared PPI networks and reference networks for structural stability under targeted deletion of nodes. For this purpose, we iteratively deleted the node with the currently highest degree from the networks (see methods for more details). We then recorded the evolution of network characteristics with progressive hub removal. The networks characteristics considered are characteristic path length, efficiency [23], diameter (length of the longest path in the network), fraction of protein pairs connected by a path, fraction of edges remaining in the network, size of the largest connected component, average component size and number of connected components. Since most of these network characteristics behave similarly as either characteristic path length or the fraction of edges remaining in the network, we focus in the following on these two characteristics. Furthermore, we analyze the number of connected components which shows a unique behavior.

#### Evolution of network characteristics

**Figure 3:**
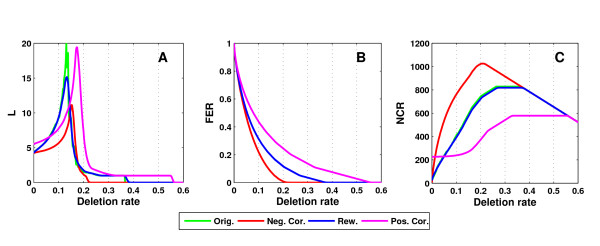
**Evolution of network characteristics**. This figure illustrates the evolution of network characteristics with increasing deletion rate for the *P. falciparum *network from LaCount et al. [7]. The development is shown for the characteristic path length *L *(**A**), the fraction of edges remaining in the network (FER) (**B**) and the number of connected components remaining (NCR) (**C**). Results for the reference networks are shown as well. In **B**, the original networks show the same behavior as the rewired networks and, thus, the corresponding green line is hidden behind the blue line for the rewired networks.

Figure 3 shows the evolution of network characteristics for the *P. falciparum *network from LaCount et al. In this case, the original network has almost the same correlation coefficient as the rewired networks, and accordingly, the evolution of network characteristics is most similar to the rewired networks. This figure illustrates the typical behavior observed for the considered network properties. With increasing deletion rate, characteristic path length increases at first up to a point after which it decreases rapidly again as the network breaks apart into isolated components (see Figure 3A). The rate of increase, the deletion rate at which the peak is observed and the rate of decrease afterwards, can differ significantly between different network types.

In general, the characteristic path length of positively correlated networks increases only slowly with increasing deletion rate and has a later and higher peak than observed for the rewired and even more so the negatively correlated references. This makes it difficult to compare the structural stability of networks by evaluating characteristic path length at one value for the deletion rate only. A small characteristic path length at one specific deletion rate does not necessarily imply tolerance to deletions. If it is due to a fragmentation of the network into isolated clusters, it actually suggests a lower tolerance.

On the other hand, the fraction of edges remaining in the network decreases continuously (see Figure 3B) until all edges are deleted. In the same way, the number of connected components increases continuously until the network consists of isolated nodes only (see Figure 3C). From this point on it decreases again. As a consequence, we can compare the structural stability easily by comparing the fraction of edges remaining (FER) in the network and the number of connected components (NCR) at a fixed deletion rate. In the following we consider a deletion rate of 10% of nodes.

#### Comparison of network stability

**Figure 4:**
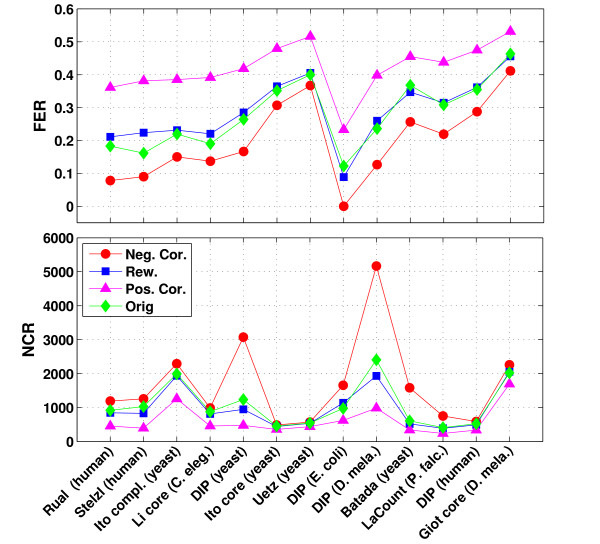
**Effects on network structure at 10% deletion rate**. The figures shows the fraction of edges remaining (FER) after deleting the 10% most highly connected nodes (top) as well as the resulting number of connected components (NCR) (bottom). Results are shown for all PPI networks examined and the corresponding reference networks and networks are sorted according to the correlation coefficient in the original PPI networks. Both network characteristics show that negatively correlated networks are most vulnerable to targeted deletion. Furthermore, the original PPI networks behave most similar to the uncorrelated references. Differences in NCR between different PPI networks are due to differences in network size.

Figure 4 illustrates for each PPI network and the corresponding reference networks the fraction of edges remaining in the network and the number of connected components after 10% of the highest connected nodes were deleted.

The fraction of edges remaining after targeted deletion is highest for the positively correlated networks and lowest for the negatively correlated networks. Thus, negative correlations in a network lead to a higher rate of interaction loss and reduce the tolerance of the network to such deletions. Again, the PPI networks have a similar or slightly smaller fraction of edges preserved than the uncorrelated networks. The correlation coefficient of a network and the fraction of edges remaining after deletion are significantly correlated (Pearson correlation of 0.72) for the protein-protein interaction networks. Nevertheless, higher correlation coefficients do not necessarily lead to a higher fraction of edges retained. For instance, although the *yeast *network by Uetz et al. and the *E. coli *network have similar correlation coefficients, the *E. coli *network and all of the corresponding reference networks have a significantly smaller fraction of edges preserved than the Uetz et al. network.

The analysis of the number of connected components leads to simular conclusions about the reduced deletion tolerance of negatively correlated networks. Although positively correlated networks consist of more connected components to begin with, the deletion of hubs results in less connected components than in the negatively correlated networks and also the rewired networks. Thus, the positively correlated networks appear to break up into isolated clusters at a much lower rate. Again we observe that the PPI networks are most similar to the uncorrelated reference networks and not to the negatively correlated ones. 

By changing the parameter *τ *(see methods) in the creation of the positively and negatively correlated reference networks, correlation coefficients can be varied significantly. We performed simulations with different values for *τ *to show that our results do not only apply to the two values chosen (see Additional File 1: Supplementary Figure 4). Furthermore, simulations were also performed for different theoretical network models with different network sizes and average degree values (see Additional File 1: Supplementary Figure 5 and Additional File 2: Supplementary Notes). These additional simulations confirm the results presented above. Thus, we can conclude that positive degree distributions increase the fragmentation of the original network but also its tolerance to targeted deletions.

## Discussion

In this article, we investigated degree correlations in protein-protein interaction networks, the associated effects on network structure and the structural stability upon selective deletion of hubs. For this purpose, we developed a model to simulate different types of degree correlations in networks. We compared several protein-protein interaction networks against negatively and positively correlated reference networks created with our model as well as the randomly correlated "null model" of Maslov and Sneppen.

Our results show that PPI networks are in general most similar to uncorrelated networks with regard to degree correlations and all other network properties considered. In this respect, they show a fairly consistent tendency across organisms and experiments. Only in a few cases did we observe considerable negative correlations such as in the complete *yeast *interaction network of Ito et al. [1]. It has been argued that the yeast-two hybrid system may artificially amplify negative degree correlations in protein-protein interaction networks [21]. Since most of the interaction networks considered for this study were determined with the Y2H approach only, our results suggest that Y2H experiments are not systematically biased towards negative degree correlations in the resulting networks. If they were, we would expect a much more obvious tendency towards negative correlations in the networks.

The differences observed in structure and stability between positively and negatively correlated networks might explain why both seem to be avoided in PPI networks. Positive degree correlations lead to a fragmentation of the network into more connected components than in the negatively correlated or uncorrelated networks. However, hubs still tend to be located in the largest component. Thus, networks with different degree correlations do not differ in the location of hubs, but in the location of small-degree nodes. In negatively correlated networks, connections between hubs and low degree nodes include these low degree nodes into the largest component. Contrary to that, in positively correlated networks the low degree nodes cluster together to form smaller connected components.

Although modularity is desired in networks to prevent unwanted cross-talk, different modules still have to interact with each other to work in a coordinated fashion. This suggests that positive degree correlations are not realized in real protein-protein interaction networks because of the consequential increased fragmentation of the network. Furthermore, positively correlated networks are characterized by short distances between hubs. As suggested by Maslov and Sneppen [18], such short distances between hubs might make the network more vulnerable to random perturbations in protein concentrations. Therefore, a separation of hubs as observed for negatively correlated networks might be favored.

On the other hand, positive degree correlations show a higher structural stability when hubs are deleted selectively. Although they consist of more components to begin with, they fall apart at a much lower rate than the other reference networks and less interactions are lost in the deletion process. In this case, the networks most affected by targeted deletion are the negatively correlated references. These differences in deletion tolerance may be explained as follows.

The deletion of a hub also removes interactions of its neighbors. As a consequence, in negatively correlated networks interactions of low degree nodes are removed, whereas in positively correlated networks interactions of other hubs are affected. However, these other hubs are most likely to be deleted in one of the next steps which would lead to the loss of the corresponding interactions anyway. Furthermore, in negatively correlated networks connections of low-degree nodes to a component are preferentially realized via hubs. Thus, the deletion of these hubs disconnects the low-degree nodes from the component. In the positively correlated networks, connections are more often via other low-degree nodes which are only deleted at a later stage.

If all interactions are more or less equally vital, the more interactions are lost the more damage is done to the cell. Furthermore, an increased disintegration of the network will prevent communication between modules and thus affect cellular processes. As a consequence, the differences in structural stability suggest a higher vulnerability of negatively correlated networks to a possible selective attack on hubs.

A biological interpretation of our results might explain why protein-protein interaction networks only show random degree correlations. Although negatively correlated networks may be more resilient to perturbations, they are also more vulnerable to a targeted attack at hubs. On the other hand, the high fragmentation of positively correlated networks might make them unfavorable as well. This suggests that both types of correlated network structures are selected against. Alternatively, network evolution processes might create more easily uncorrelated structures. In this case, positive or negative degree correlations may not be beneficial enough to lead to a deviation from these processes.

## Conclusion

In this article, we showed that apart from the degree distribution, degree correlations can have a significant effect on network structure and the stability of the network under selective deletion of hubs. We observed that positive degree correlations lead to an increased fragmentation of the network into isolated components. Negative correlations, on the other hand, decrease the tolerance of the network to a selective deletion of hubs. Interestingly, we found for the PPI networks that they deviate only marginally from the uncorrelated "null model" both with respect to degree correlations and tolerance to targeted deletions. Thus, although large variations are possible, they are not realized at all in biological interaction networks. This may be explained by selective disadvantages associated with both types of degree correlations under different conditions.

## Methods

### Uncorrelated (rewired) reference networks

Uncorrelated reference networks (the "null model") are generated using the rewiring method by Maslov and Sneppen [18]. They show only degree correlations which are random given the degree distribution. One edge rewiring step consists in choosing two random edges (*u*, *v*) and (*w*, *x*) and replacing them by the edges (*u*, *x*) and (*w*, *v*) if none of the edges already exists. Further restrictions have to be imposed if not all edges are possible, e.g. if the network under consideration was created in a large-scale experiment with different sets of baits and preys. In this case, edges between two preys are not allowed. This edge-swapping strategy is repeated a sufficiently large number of times to completely randomize any correlation between degree values of connected nodes.

### Negatively and positively correlated reference networks

For both types of reference networks, we start with a network containing only the nodes but not the edges of the original network. Each node is then assigned its degree value in the original network. We then choose iteratively the node *v *with the highest assigned degree whose current degree is lower than this assigned degree. Edges are added to this node until its degree value matches its original degree value. To add an edge a random node *u *is chosen from the remaining nodes with probability P(u)~kuτ
 MathType@MTEF@5@5@+=feaafiart1ev1aaatCvAUfKttLearuWrP9MDH5MBPbIqV92AaeXatLxBI9gBaebbnrfifHhDYfgasaacH8akY=wiFfYdH8Gipec8Eeeu0xXdbba9frFj0=OqFfea0dXdd9vqai=hGuQ8kuc9pgc9s8qqaq=dirpe0xb9q8qiLsFr0=vr0=vr0dc8meaabaqaciaacaGaaeqabaqabeGadaaakeaacqWGqbaucqGGOaakcqWG1bqDcqGGPaqkcqGG+bGFcqWGRbWAdaqhaaWcbaGaemyDauhabaacciGae8hXdqhaaaaa@3749@.

To create negatively correlated reference networks *τ *was set to 0. As a consequence, each node is chosen with equal probability. Since low-degree nodes are most abundant, hubs will then be connected preferentially to low-degree nodes.

For positively correlated references *τ *was set to 3. As a consequence, high-degree nodes are chosen with higher probability and connections between hubs are increased.

### Skewness of a distribution

Although there exist several alternative definitions of skewness, the one most commonly used is

skewness=∑v∈V(kv−k¯)3(|V|−1)s3
 MathType@MTEF@5@5@+=feaafiart1ev1aaatCvAUfKttLearuWrP9MDH5MBPbIqV92AaeXatLxBI9gBaebbnrfifHhDYfgasaacH8akY=wiFfYdH8Gipec8Eeeu0xXdbba9frFj0=OqFfea0dXdd9vqai=hGuQ8kuc9pgc9s8qqaq=dirpe0xb9q8qiLsFr0=vr0=vr0dc8meaabaqaciaacaGaaeqabaqabeGadaaakeaacqWGZbWCcqWGRbWAcqWGLbqzcqWG3bWDcqWGUbGBcqWGLbqzcqWGZbWCcqWGZbWCcqGH9aqpdaWcaaqaamaaqababaGaeiikaGIaem4AaS2aaSbaaSqaaiabdAha2bqabaGccqGHsislcuWGRbWAgaqeaiabcMcaPmaaCaaaleqabaGaeG4mamdaaaqaaiabdAha2jabgIGiolabdAfawbqab0GaeyyeIuoaaOqaamaabmaabaWaaqWaaeaacqWGwbGvaiaawEa7caGLiWoacqGHsislcqaIXaqmaiaawIcacaGLPaaacqWGZbWCdaahaaWcbeqaaiabiodaZaaaaaaaaa@518E@

where *V *is the set of nodes in the network, *k*_*v *_the degree of node *v*, k¯
 MathType@MTEF@5@5@+=feaafiart1ev1aaatCvAUfKttLearuWrP9MDH5MBPbIqV92AaeXatLxBI9gBaebbnrfifHhDYfgasaacH8akY=wiFfYdH8Gipec8Eeeu0xXdbba9frFj0=OqFfea0dXdd9vqai=hGuQ8kuc9pgc9s8qqaq=dirpe0xb9q8qiLsFr0=vr0=vr0dc8meaabaqaciaacaGaaeqabaqabeGadaaakeaacuWGRbWAgaqeaaaa@2E23@ the average degree of the network and *s *the sample standard deviation of the degree distribution. For symmetric distributions the skewness is close to zero whereas for left-tailed distributions it is negative and for right-tailed distributions, such as e.g. power-law distributions, it is positive.

### Evaluation of degree correlations

To quantify degree correlations in a network we use Pearson's correlation coefficient *r *between degree values of connected nodes calculated over all edges in the network. Here, undirected edges are treated as two directed edges. Let (*e*_1_, ... *e*_*m*_) be the vector of all edges in the set of undirected edges and *k*_*v *_the degree of a node *v*. Then we set *x *and *y *as two vectors of length 2*m *with *x*_2*i*-1 _= *k*_*u*_, *x*_2*i *_= *k*_*v*_, *y*_2*i*-1 _= *x*_2*i *_and *y*_2*i *_= *x*_2*i*-1 _for *e*_*i *_= (*u*, *v*). The correlation coefficient *r *is then defined as

r=∑i=12m(xi−x¯)(yi−y¯)(2m−1)sxsy
 MathType@MTEF@5@5@+=feaafiart1ev1aaatCvAUfKttLearuWrP9MDH5MBPbIqV92AaeXatLxBI9gBaebbnrfifHhDYfgasaacH8akY=wiFfYdH8Gipec8Eeeu0xXdbba9frFj0=OqFfea0dXdd9vqai=hGuQ8kuc9pgc9s8qqaq=dirpe0xb9q8qiLsFr0=vr0=vr0dc8meaabaqaciaacaGaaeqabaqabeGadaaakeaacqWGYbGCcqGH9aqpdaWcaaqaamaaqadabaGaeiikaGIaemiEaG3aaSbaaSqaaiabdMgaPbqabaGccqGHsislcuWG4baEgaqeaiabcMcaPiabcIcaOiabdMha5naaBaaaleaacqWGPbqAaeqaaOGaeyOeI0IafmyEaKNbaebacqGGPaqkaSqaaiabdMgaPjabg2da9iabigdaXaqaaiabikdaYiabd2gaTbqdcqGHris5aaGcbaGaeiikaGIaeGOmaiJaemyBa0MaeyOeI0IaeGymaeJaeiykaKIaem4Cam3aaSbaaSqaaiabdIha4bqabaGccqWGZbWCdaWgaaWcbaGaemyEaKhabeaaaaaaaa@5171@

with x¯
 MathType@MTEF@5@5@+=feaafiart1ev1aaatCvAUfKttLearuWrP9MDH5MBPbIqV92AaeXatLxBI9gBaebbnrfifHhDYfgasaacH8akY=wiFfYdH8Gipec8Eeeu0xXdbba9frFj0=OqFfea0dXdd9vqai=hGuQ8kuc9pgc9s8qqaq=dirpe0xb9q8qiLsFr0=vr0=vr0dc8meaabaqaciaacaGaaeqabaqabeGadaaakeaacuWG4baEgaqeaaaa@2E3D@ and y¯
 MathType@MTEF@5@5@+=feaafiart1ev1aaatCvAUfKttLearuWrP9MDH5MBPbIqV92AaeXatLxBI9gBaebbnrfifHhDYfgasaacH8akY=wiFfYdH8Gipec8Eeeu0xXdbba9frFj0=OqFfea0dXdd9vqai=hGuQ8kuc9pgc9s8qqaq=dirpe0xb9q8qiLsFr0=vr0=vr0dc8meaabaqaciaacaGaaeqabaqabeGadaaakeaacuWG5bqEgaqeaaaa@2E3F@ the sample means and *s*_*x *_and *s*_*y *_the sample standard deviations of *x *and *y*. Positive values of *r *indicate a positive correlation and negative values a negative correlation between the degrees of associated nodes. If the degree distribution is skewed to the right, we observe negative correlation coefficients even for the uncorrelated reference networks.

### Simulation of measurement errors

To simulate the effects of measurement errors on degree correlations and network stability, 10% of the edges were removed randomly and replaced by the same number of edges. Here, four strategies were applied. First, 10% of the edges were rewired as described for the "null model". Second and third, edges were added using the method for creating either negatively or positively correlated networks after removing only 10% and not all edges from the starting network. And fourth, the preferential attachment method described in [15] was used. In this case, two nodes *u *and *v *are connected by a wrong interaction with probability

θ(kv+ι)(ku+ι)∑w∈V(kw+ι).
 MathType@MTEF@5@5@+=feaafiart1ev1aaatCvAUfKttLearuWrP9MDH5MBPbIqV92AaeXatLxBI9gBaebbnrfifHhDYfgasaacH8akY=wiFfYdH8Gipec8Eeeu0xXdbba9frFj0=OqFfea0dXdd9vqai=hGuQ8kuc9pgc9s8qqaq=dirpe0xb9q8qiLsFr0=vr0=vr0dc8meaabaqaciaacaGaaeqabaqabeGadaaakeaaiiGacqWF4oqCdaWcaaqaaiabcIcaOiabdUgaRnaaBaaaleaacqWG2bGDaeqaaOGaey4kaSIae8xUdKMaeiykaKIaeiikaGIaem4AaS2aaSbaaSqaaiabdwha1bqabaGccqGHRaWkcqWF5oqAcqGGPaqkaeaadaaeqaqaaiabcIcaOiabdUgaRnaaBaaaleaacqWG3bWDaeqaaOGaey4kaSIae8xUdKMaeiykaKcaleaacqWG3bWDcqGHiiIZcqWGwbGvaeqaniabggHiLdaaaOGaeiOla4caaa@4B52@

Here *θ *controls the error rate and is tuned such that approximately 10% wrong interactions are added. *l *is used as a pseudocount and set to 1.

### Characteristic path length

Characteristic path length *L *is defined as average shortest path length over all pairs of nodes between which a path actually exists [24]. Unconnected pairs are not taken into consideration here. For the calculation of characteristic path length between hubs, only shortest paths between hubs were considered. However, these shortest paths can also pass non-hub nodes. Hubs are defined as the top 10% of nodes with the highest degree. Calculation of shortest path is done by breadth-first searches starting from each node on the unweighted interaction graph.

### Targeted deletion of nodes

Targeted deletion of hubs is simulated by iteratively deleting the node with the currently highest degree from the network. This is contrary to random failure for which nodes are deleted randomly without regard to their degree. For our purposes, nodes were not deleted with decreasing order of their degree in the original network as described by Albert et al. [17]. Instead, we recalculate the node degrees at each step and then delete the node with the currently highest degree. This different approach is used to avoid an artificial advantage for positively correlated networks. Since hubs are preferentially connected to other hubs in these networks, the deletion of some of the hubs will preferentially decrease the degree of other hubs. Consequently, some of the nodes which were hubs in the original network might no longer be hubs after a few node deletion steps. Therefore, it is more appropriate to delete the nodes which then have the highest degree.

## Authors' contributions

CF implemented the simulation programs and analyzed the results. RZ participated in the design of the study and interpreting the results. The manuscript was written by CF and RZ. All authors read and approved the final manuscript.

## Supplementary Material

Additional file 1 Supplementary Figures. This file contains additional figures illustrating the results for the PPI networks and reference networks. Furthermore results are shown for reference networks created with different values of *τ *and for theoretical network models. Click here for file

Additional file 2 Supplementary Notes. This file contains supplementary notes explaining simulations and results for theoretical network models. Click here for file
