# Supplementary material for: Influence of degree correlations on network structure and stability in protein-protein interaction networks
Source: BMC Bioinformatics. 2007 Aug 9;8:297. doi: 10.1186/1471-2105-8-297 (PMC1995226; doi:10.1186/1471-2105-8-297)
Supplement: Additional file 1 — Supplementary Figures. This file contains additional figures illustrating the results for the PPI networks and reference networks. Furthermore results are shown for reference networks created with different values of τ and for theoretical network models. [file 1471-2105-8-297-S1.pdf]

Supplementary Figures

Supplementary Figure 1 - Degree distributions of experimental protein-protein interaction networks

This figure shows the degree distribution for the experimental PPI networks which are sorted according to the correlation coefficient between degrees of connected nodes. From top left to bottom right we have the *human* Y2H interaction networks from Rual et al. [1] and Stelzl et al. [2], the complete *yeast* Y2H network from Ito et al. [3], the *C.elegans* core Y2H network from Li et al. [4], the *yeast* interaction network from DIP [5], the core *yeast* network from Ito et al., the *yeast* Y2H network from Uetz et al. [6], the *E. Coli* network from DIP, the *drosophila* network from DIP, the high confidence *yeast* interaction network compiled by Batada et al. [7], the *P. falciparum* Y2H network from LaCount et al. [8], the *human* interacton network from DIP and the *drosophila* core Y2H interaction network from Giot et al. [9].

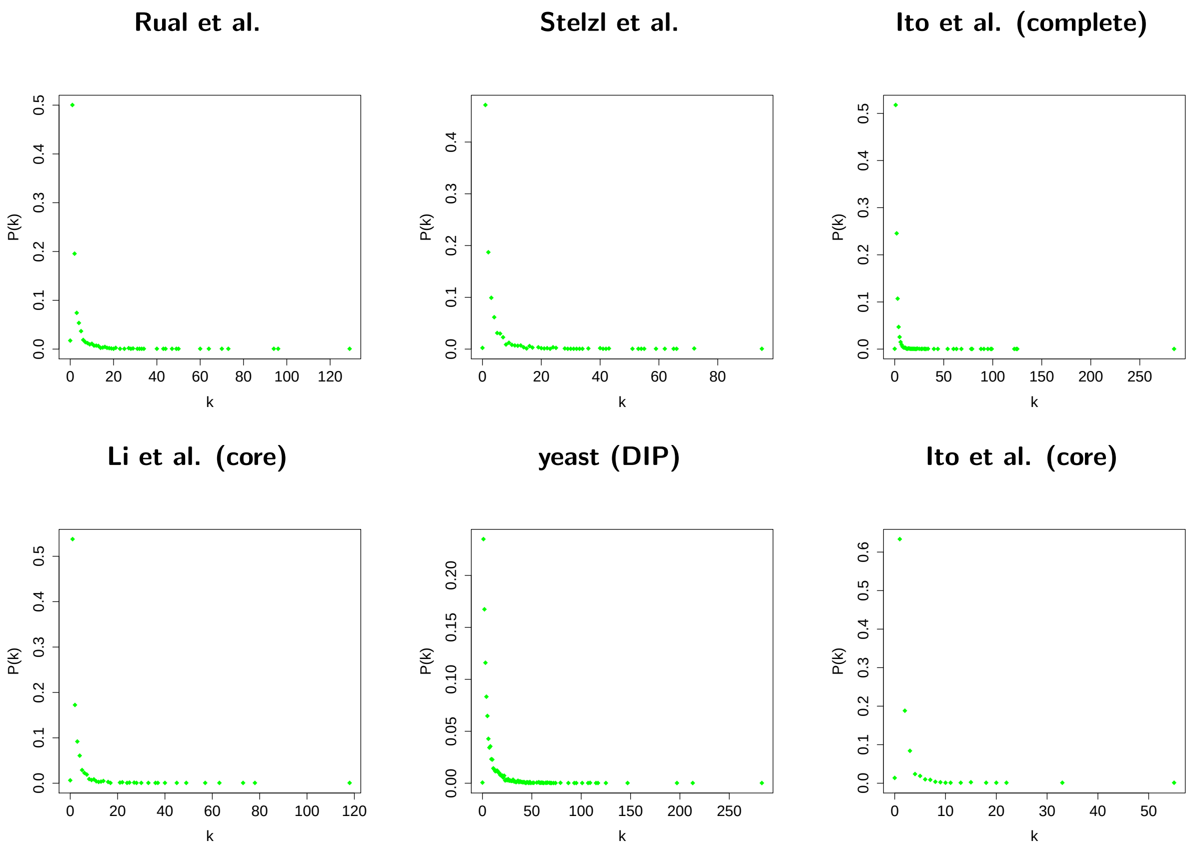

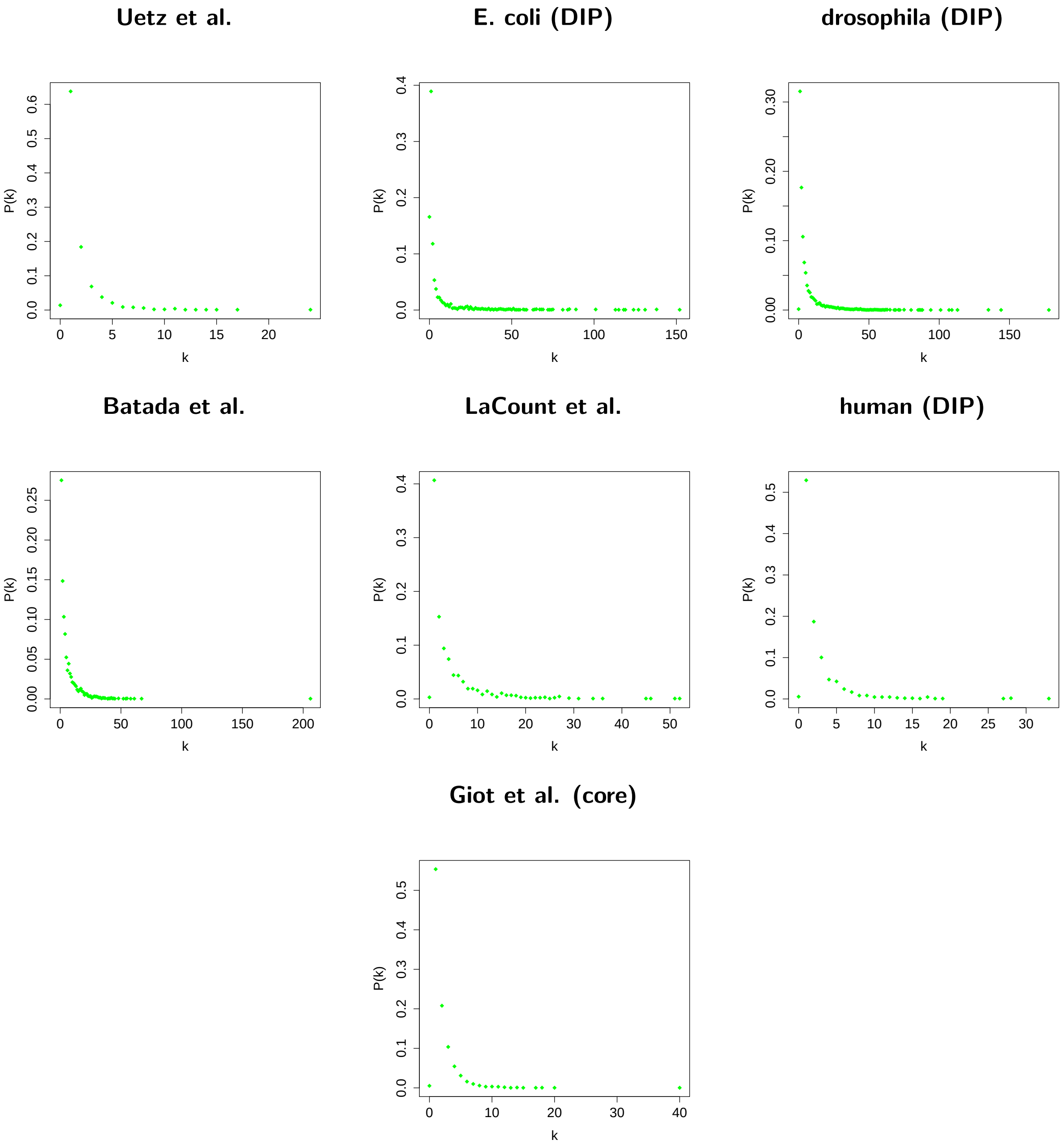
Uetz et al.
E. coli (DIP)
drosophila (DIP)
Batada et al.
LaCount et al.
human (DIP)
Giot et al. (core)
P(k)
k

Supplementary Figure 2 - Simulation of measurement errors

Simulations on the effect of measurement errors were performed by deleting randomly 10% of edges and introducing randomly the same number of edges again. For this purpose, four types of error mechanisms were used: a) 10% of the edges were rewired as described for the "null model" (Rew. Error); b) and c) edges were added using the method for creating negatively (Neg. Cor. Error) and positively (Pos. Cor. Error) correlated networks after removing only 10% of the edges from the starting network; d) the preferential attachment scenario described in [10] was applied (Pref. Attach. Error). The figures show the results for each of the 100 simulation runs for each error mechanism as well as the average results for the original and reference networks. On the x coordinate the correlation coefficients of the networks are shown and on the y coordinate the fraction of edges remaining in the network at 10% deletion rate.

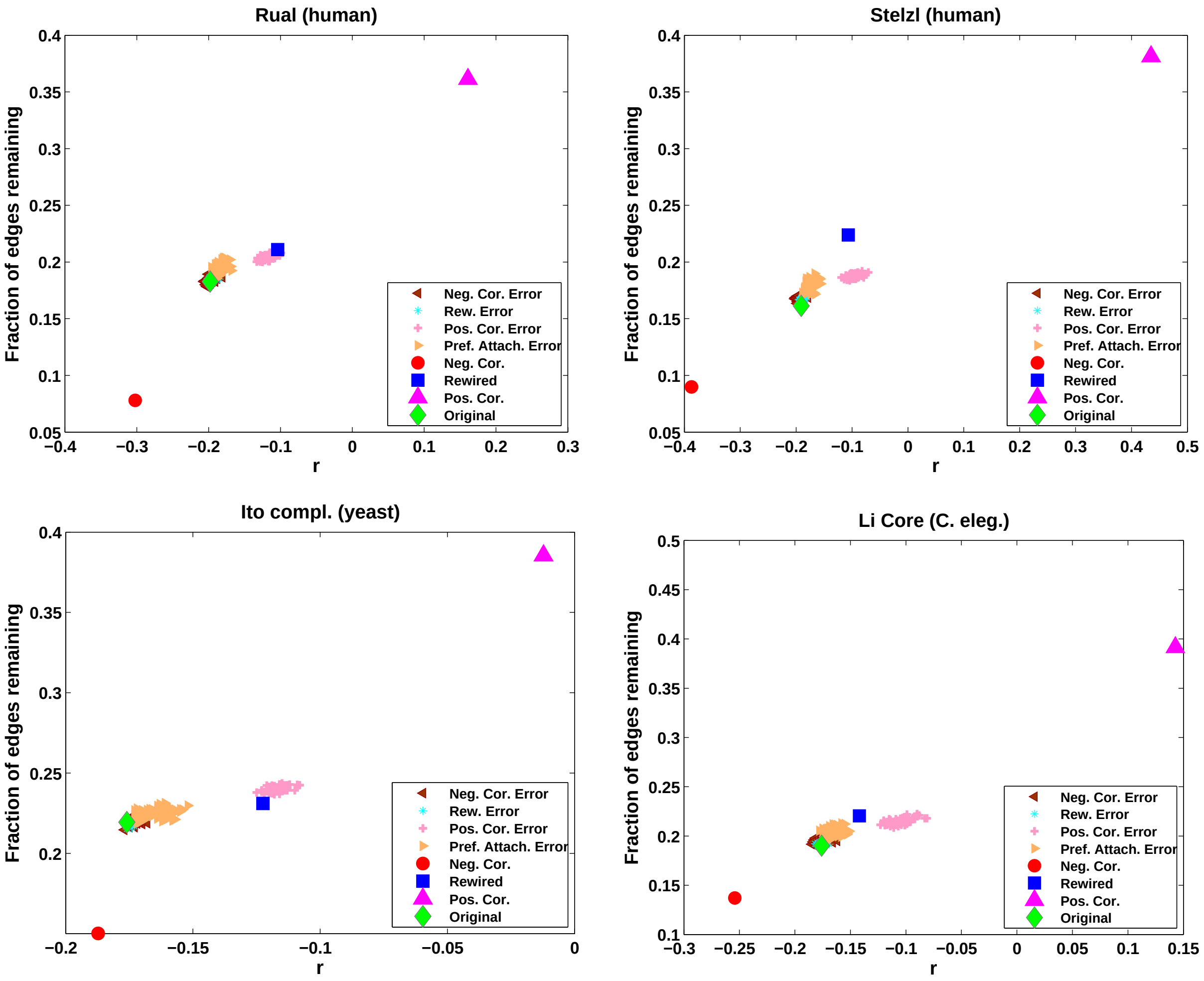

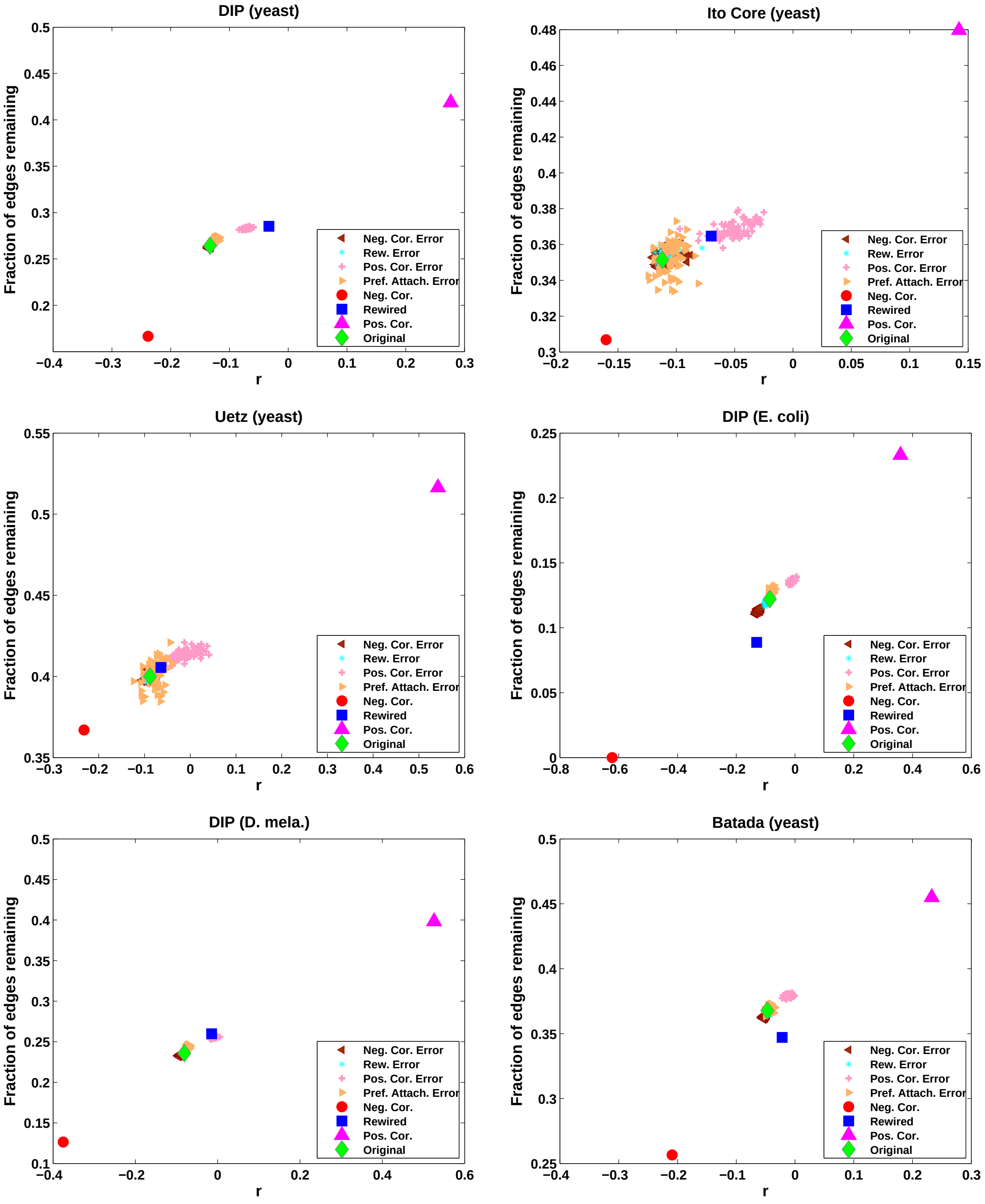
DIP (yeast)
Ito Core (yeast)
Uetz (yeast)
DIP (E. coli)
DIP (D. mela.)
Batada (yeast)
Fraction of edges remaining
r
Neg. Cor. Error
Rew. Error
Pos. Cor. Error
Pref. Attach. Error
Neg. Cor.
Rewired
Pos. Cor.
Original

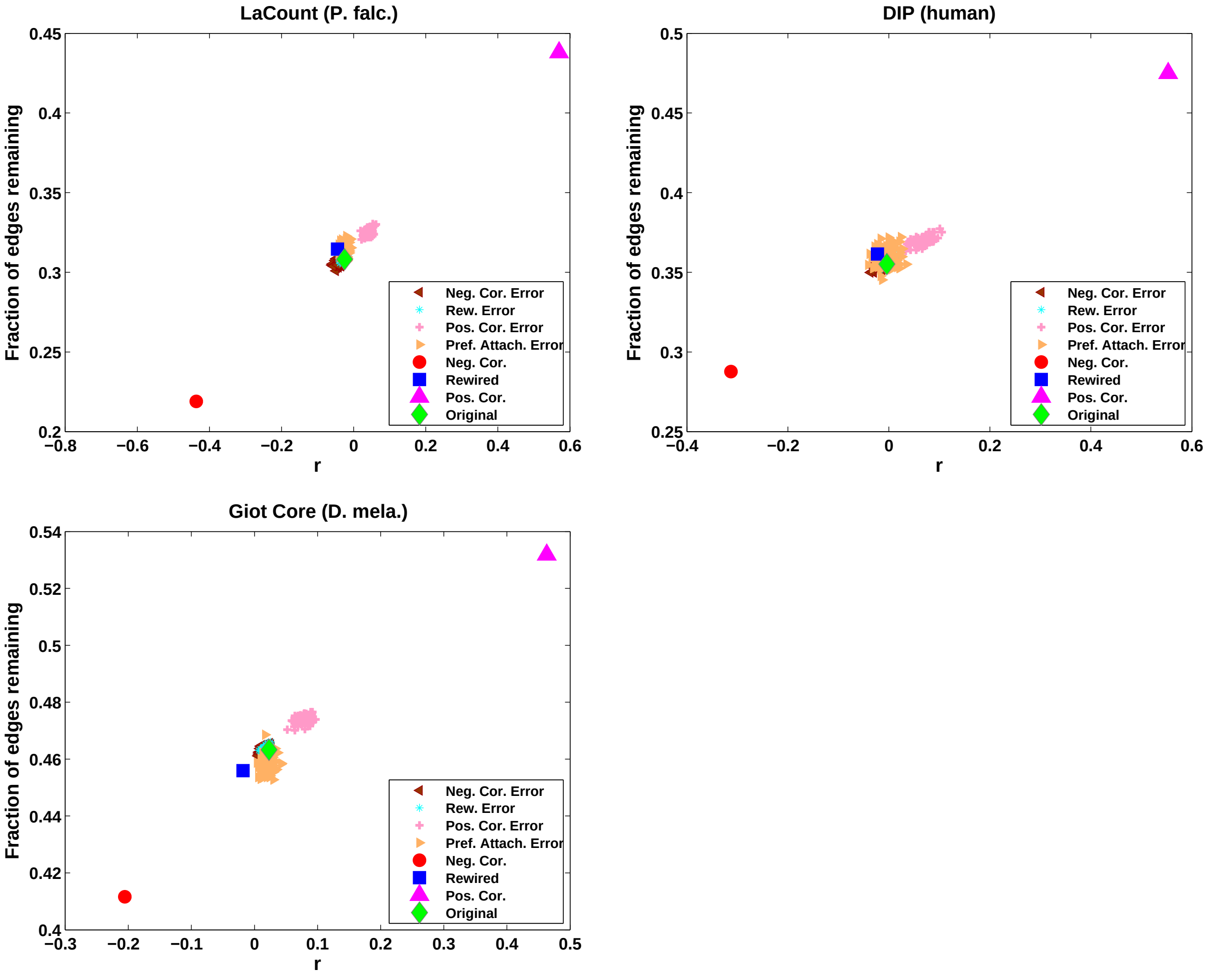
LaCount (P. falc.)
DIP (human)
Giot Core (D. mela.)
Fraction of edges remaining
r
Neg. Cor. Error
Rew. Error
Pos. Cor. Error
Pref. Attach. Error
Neg. Cor.
Rewired
Pos. Cor.
Original

Supplementary Figure 3 - Characteristic path length

Characteristic path length is shown for all nodes (top) and for hubs only (bottom) for experimental PPI networks and corresponding reference models. Networks are sorted according to correlation coefficient as described in Figure 1. No consistent tendency is observed for overall characteristic path length. If we restrict the calculation to hubs only, we observe that, as expected, the characteristic path length is longest in negatively correlated networks and shortest in positively correlated networks.

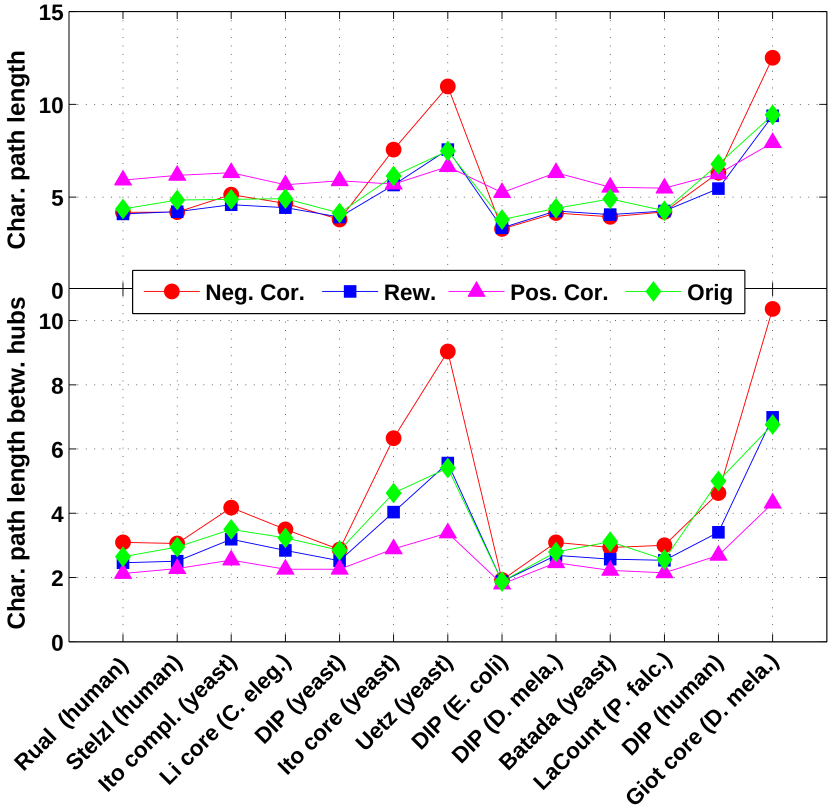

Supplementary Figure 4 - Effect of degree correlations

Simulations were performed with reference networks created with different values of the parameter τ (13 different values between -0.3 and 5 were chosen). The following figure shows for each of the reference networks on the x coordinate the correlation coefficient and on the y coordinate the number of connected components in the original reference network, the fraction of edges remaining at a deletion rate of 10% and the number of connected components at this deletion rate, respectively . In each case, the left hand side figure shows the results for the networks of Rual et al., Stelzl et al., Ito et al. (complete), Li et al. (core), *yeast* (DIP), Ito et al. (core) and Uetz et al. The right hand side shows the results for the networks for *E. coli* (DIP), *D. mela.* (DIP), Batada et al., LaCount et al., *human* (DIP) and Giot et al. (core).

The results for different values of τ for each PPI network are connected by lines to show the overall tendency. The number of connected components in the original reference networks as well as the fraction of edges remaining after 10% deletion rate increase continously with the correlation coefficient. The number of connected components at 10% deletion rate on the other hand decreases with the correlation coefficient. However, for high correlation coefficients saturation effects are observed which lead to a minor increase again. Nevertheless, even in this case the values observed are smaller than for the negatively correlated networks

Number of connected components

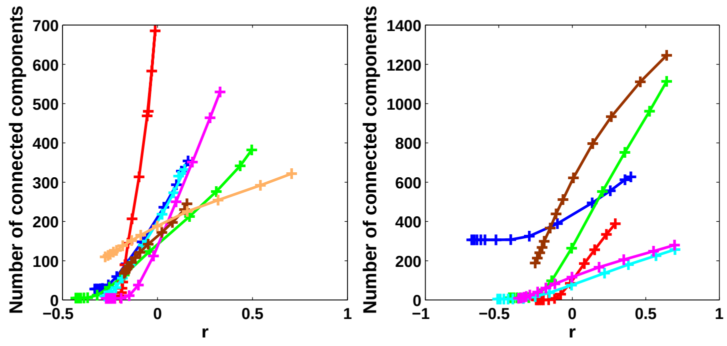

Fraction of edges remaining at 10% deletion rate

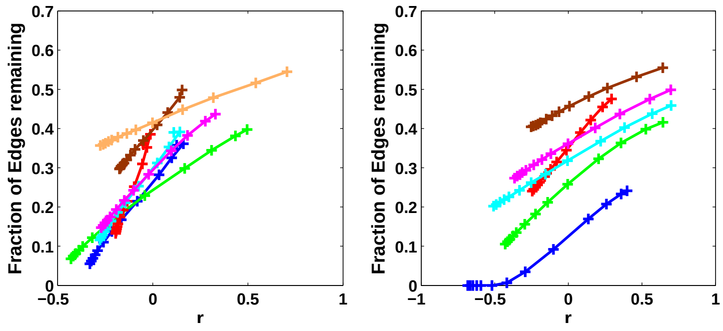

Number of connected components at 10% deletion rate

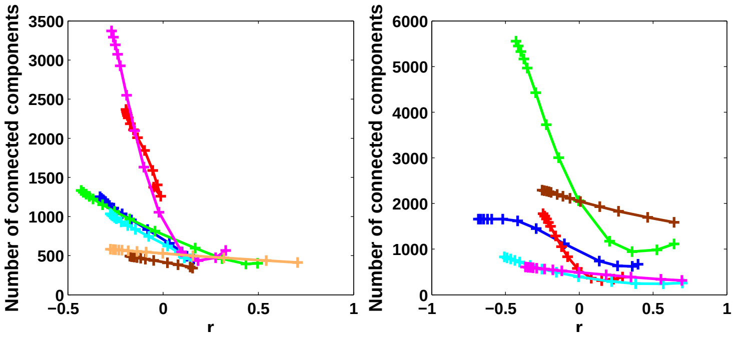

Supplementary Figure 5 - Results for theoretical network models

This figure shows the results for the theoretical network models: Erdős and Rényi (ER) random graphs [11], exponential (EX) networks and power-law (PL) networks. Network sizes of 200, 1000 and 5000 were simulated to compare the effect of network size. Average degree values $\bar{k}$ of 2, 4, and 6 were simulated. Results are shown for the correlation coefficients, the number of connected components in the original network, the fraction of edges remaining in the network at 10% deletion rate and the number of connected components at this deletion rate. Both original number of connected components and the number of connected components at 10% deletion rate were normalized by dividing by the network size of the original networks.

Correlation coefficient

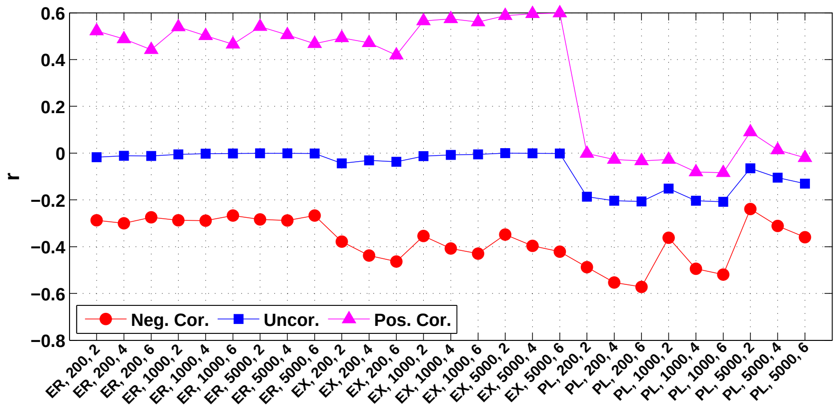

Number of connected components before deletion

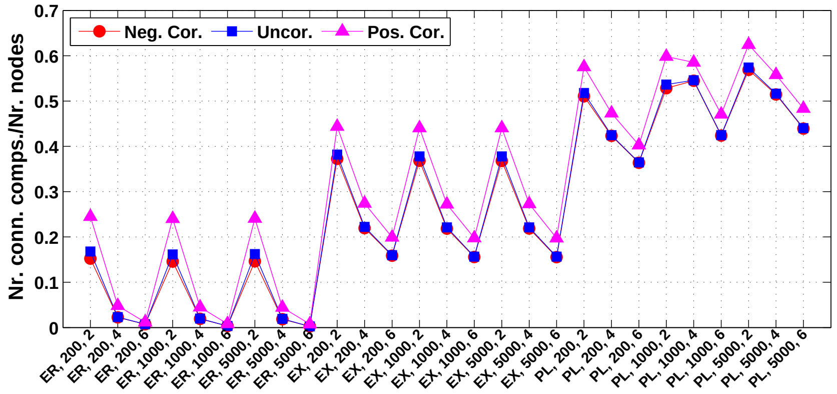

Fraction of edges remaining at 10% deletion rate

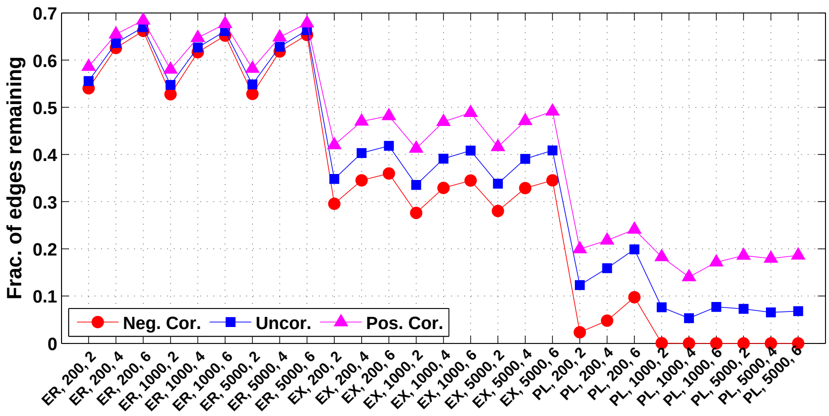

Number of connected components at 10% deletion rate

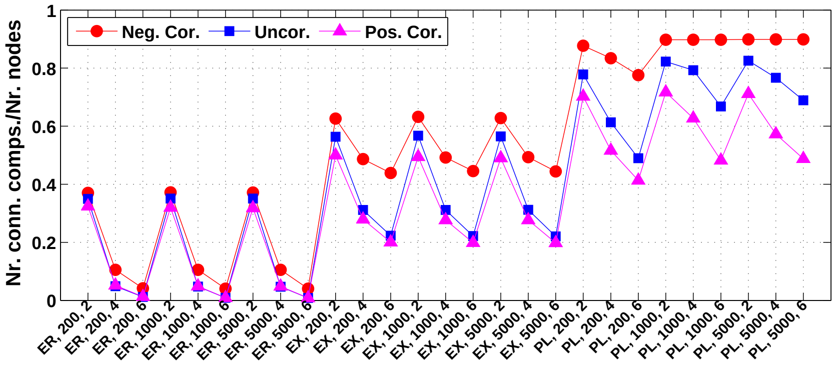

References

[1] Rual JF, Venkatesan K, Hao T, Hirozane-Kishikawa T, Dricot A, Li N, Berriz GF, Gibbons FD, Dreze M, Ayivi-Guedehoussou N, Klitgord N, Simon C, Boxem M, Milstein S, Rosenberg J, Goldberg DS, Zhang LV, Wong SL, Franklin G, Li S, Albala JS, Lim J, Fraughton C, Llamosas E, Cevik S, Bex C, Lamesch P, Sikorski RS, Vandenhaute J, Zoghbi HY, Smolyar A, Bosak S, Sequerra R, Doucette-Stamm L, Cusick ME, Hill DE, Roth FP, Vidal M: **Towards a proteome-scale map of the human protein-protein interaction network.** *Nature* 2005, **437**:1173–8.

[2] Stelzl U, Worm U, Lalowski M, Haenig C, Brembeck FH, Goehler H, Stroedicke M, Zenkner M, Schoenherr A, Koeppen S, Timm J, Mintzlaff S, Abraham C, Bock N, Kietzmann S, Goedde A, Toksz E, Droege A, Krobitsch S, Korn B, Birchmeier W, Lehrach H, Wanker EE: **A human protein-protein interaction network: a resource for annotating the proteome.** *Cell* 2005, **122**:957–68.

[3] Ito T, Chiba T, Ozawa R, Yoshida M, Hattori M, Sakaki Y: **A comprehensive two-hybrid analysis to explore the yeast protein interactome.** *Proc Natl Acad Sci USA* 2001, **98**:4569–74.

[4] Li S, Armstrong CM, Bertin N, Ge H, Milstein S, Boxem M, Vidalain PO, Han JDJ, Chesneau A, Hao T, Goldberg DS, Li N, Martinez M, Rual JF, Lamesch P, Xu L, Tewari M, Wong SL, Zhang LV, Berriz GF, Jacotot L, Vaglio P, Reboul J, Hirozane-Kishikawa T, Li Q, Gabel HW, Elewa A, Baumgartner B, Rose DJ, Yu H, Bosak S, Sequerra R, Fraser A, Mango SE, Saxton WM, Strome S, Heuvel SVD, Piano F, Vandenhaute J, Sardet C, Gerstein M, Doucette-Stamm L, Gunsalus KC, Harper JW, Cusick ME, Roth FP, Hill DE, Vidal M: **A map of the interactome network of the metazoan C. elegans.** *Science* 2004, **303**:540–3.

[5] Xenarios I, Salwínski L, Duan XJ, Higney P, Kim SM, Eisenberg D: **DIP, the Database of Interacting Proteins: a research tool for studying cellular networks of protein interactions.** *Nucleic Acids Res* 2002, **30**:303–5.

[6] Uetz P, Giot L, Cagney G, Mansfield TA, Judson RS, Knight JR, Lockshon D, Narayan V, Srinivasan M, Pochart P, Qureshi-Emili A, Li Y, Godwin B, Conover D, Kalbfleisch T, Vijayadamodar G, Yang M, Johnston M, Fields S, Rothberg JM: **A comprehensive analysis of protein-protein interactions in Saccharomyces cerevisiae.** *Nature* 2000, **403**:623–7.

[7] Batada NN, Reguly T, Breitkreutz A, Boucher L, Breitkreutz BJ, Hurst LD, Tyers M: **Stratus not altocumulus: a new view of the yeast protein interaction network.** *PLoS Biol* 2006, **4**(10):e317.

[8] LaCount DJ, Vignali M, Chettier R, Phansalkar A, Bell R, Hesselberth JR, Schoenfeld LW, Ota I, Sahasrabudhe S, Kurschner C, Fields S, Hughes RE: **A protein interaction network of the malaria parasite Plasmodium falciparum.** *Nature* 2005, **438**:103–7.

[9] Giot L, Bader JS, Brouwer C, Chaudhuri A, Kuang B, Li Y, Hao YL, Ooi CE, Godwin B, Vitols E, Vijayadamodar G, Pochart P, Machineni H, Welsh M, Kong Y, Zerhusen B, Malcolm R, Varrone Z, Collis A, Minto M, Burgess S, McDaniel L, Stimpson E, Spriggs F, Williams J, Neurath K, Ioime N, Agee M, Voss E, Furtak K, Renzulli R, Aanensen N, Carrolla S, Bickelhaupt E, Lazovatsky Y, DaSilva A, Zhong J, Stanyon CA, Finley RL, White KP, Braverman M, Jarvie T, Gold S, Leach M, Knight J, Shimkets RA, McKenna MP, Chant J, Rothberg JM: **A protein interaction map of Drosophila melanogaster.** *Science* 2003, **302**:1727–36.

[10] Friedel CC, Zimmer R: **Inferring topology from clustering coefficients in protein-protein interaction networks.** *BMC Bioinformatics* 2006, **7**:519.

[11] Erdős P, Rényi A: **On random graphs**. *Publicationes Mathematicae* 1959, **6**:290–297.
