# Supplementary material for: Influence of degree correlations on network structure and stability in protein-protein interaction networks
Source: BMC Bioinformatics. 2007 Aug 9;8:297. doi: 10.1186/1471-2105-8-297 (PMC1995226; doi:10.1186/1471-2105-8-297)
Supplement: Additional file 2 — Supplementary Notes. This file contains supplementary notes explaining simulations and results for theoretical network models. [file 1471-2105-8-297-S2.pdf]

Supplementary Notes

Generation of theoretical network models

The influence of degree correlations on the network structure and tolerance to selective deletion of hubs was also analyzed for theoretical network models. For our purposes, three types of network models were generated: Erdős and Rényi random graphs (ER), exponential networks (EX) and power-law networks (PL).

Network sizes of 200, 1000 and 5000 were chosen to evaluate the effects of different network sizes. They are meant to represent very small networks (such as virus PPI networks), medium and larger networks. We found that network size mostly affects how much variations are observed. For 200 nodes, variations were considerable. for 5000 nodes only very slight variations were observed. Thus, no simulations were performed for networks larger than this. Furthermore, different average degree values (2, 4 and 6) were considered.

Random graphs

Erdős and Rényi random graphs [1] (ER) are created using the following simple model [2]. In a network containing n nodes, each pair of nodes is connected with probability p. The distribution of node degrees in random graphs follows a Poisson distribution and the expected clustering coefficients is p.

Exponential and power-law networks

Exponential networks (EX) are characterized by an exponential distribution of degree values such that $P(k) = \lambda e^{-\lambda k}$ for some constant λ. In power-law networks (PL) the probability of a node having degree k is proportional to $k^{-\gamma}$ for some constant γ. For our purposes, random networks with a given degree distribution are generated using the method described by Chung and Lu [3]. First an expected degree sequence $\mathbf{w} = (w_1, \ldots, w_n)$ is given for the n nodes which follows the chosen distribution. Then an edge is created between nodes v_i and v_j with probability $p_{ij} = w_i w_j \rho$ with $\rho = (1/\sum_i w_i)$. The w_i for the power-law networks are generated with an exponential cut-off as described by Newman *et al.* [4] using a two-step transformation/rejection method.

Simulation results

Simulation results for the theoretical networks are shown in Additional File 2: Supplementary Figure 5. The same observations could be made as for the protein-protein interaction networks. Positive correlations

in networks lead to a fragmentation of the network into more connected components. On the other hand, more edges are lost in negatively corelated networks after the selective deletion of hubs and the number of connected components is higher after the deletions than in the positively correlated networks.

References

1. Erdős P, Rényi A: **On random graphs**. *Publicationes Mathematicae* 1959, **6**:290–297.
2. Bollobás B: *Random Graphs*. Cambridge University Press 2001.
3. Chung F, Lu L: **The average distances in random graphs with given expected degrees.** *Proc Natl Acad Sci USA* 2002, **99**:15879–82.
4. Newman MEJ, Strogatz SH, Watts DJ: **Random graphs with arbitrary degree distributions and their applications**. *Phys. Rev. E* 2001, **64**(2):026118.
